# Mortality Statistics

**Published:** 1874-12

**Authors:** Ben. C. Miller

**Affiliations:** Sanitary Superintendent


***Chicago Mortality Report for October, 1874.*** Reported by Dr. BEN. C. MILLER, Sanitary Superintendent.

MORTALITY IN MONTH OF OCTOBER, 1874.

| | |
|---|---|
| Accident, by drowning | 3 |
| " by fall | 4 |
| " in planing mill | 1 |
| " run over by bus | 1 |
| " run over by buggy | 1 |
| " by shooting | 1 |
| " by railroad | 3 |
| Abscess, pelvic | 2 |
| Apoplexy | 8 |
| Asphyxia | 1 |
| Asthma | 1 |
| Ataxia, locomotor | 1 |
| Ascites | 4 |
| Atelectasis pulmonum | 1 |
| Bowels, perforation of | 1 |
| Brain, congestion of | 8 |
| " inflammation of | 3 |
| " softening of | 3 |
| Bronchitis | 5 |
| " capillary | 6 |
| Cancer | 1 |
| " of breast | 1 |
| " of liver | 1 |
| " of uterus | 4 |
| Cholera infantum | 34 |
| " morbus | 1 |
| Consumption | 63 |
| Convulsions | 74 |
| " epileptic | 1 |
| Croup | 9 |
| " membranous | 2 |
| Cyanosis | 2 |
| Debility, general | 7 |
| Delirium tremens | 3 |
| Diabetis | 1 |
| Diarrhœa | 17 |
| " chronic | 5 |
| Diphtheria | 10 |
| Dropsy, general | 4 |
| " abdominal | 1 |
| Dysentery | 11 |
| Dyspepsia | 1 |
| Enlargement of bronchial glands | 1 |
| " of embolia | 1 |
| Enteritis | 11 |
| Entero-colitis | 8 |
| Epilepsy | 1 |
| Exposure | 1 |
| Erysipelas | 2 |
| Exhaustion | 1 |
| Fever, puerperal | 1 |
| " remittent | 1 |
| " scarlet | 6 |
| " typhoid | 33 |
| Fungus hematodes | 1 |
| Gastritis | 3 |
| Gastro enteritis | 3 |
| Hemorrhagica purpura | 1 |
| Heart, disease of | 4 |
| " congestion of | 1 |
| " dropsy of | 1 |
| " organic disease of | 1 |
| " mitral valvular disease of | 1 |
| " valvular disease of | 3 |

| | |
|---|---|
| Hip, disease of | 1 |
| Hydropericarditis | 10 |
| Inanition | 14 |
| Intemperance | 2 |
| Intussusception | 1 |
| Icterus | 1 |
| Jaundice | 2 |
| Kidneys, Bright's disease | 3 |
| " inflammation of | 1 |
| Liver, disease of | 1 |
| " cirrhosis of | 1 |
| Lungs, congestion of | 3 |
| " gangrene of | 1 |
| " hemorrhage of | 1 |
| " œdema of | 1 |
| Meningitis | 8 |
| " cerebro-spinal | 4 |
| " tubercular | 2 |
| Neglect at birth | 1 |
| Old age | 18 |
| Paralysis | 4 |
| Pericarditis | 1 |
| Peritonitis | 3 |
| Pneumonia | 23 |
| " typhoid | 5 |
| Pleurisy | 2 |
| Pharyngitis | 1 |
| Scrofula | 1 |
| Septicæmia, puerperal | 1 |
| Shock, following surgical operation | 1 |
| Stomach, ulceration of | 2 |
| Syphilis | 2 |
| Tabes mesenterica | 22 |
| Teething | 9 |
| Trismus | 5 |
| Tonsilitis | 2 |
| Suicides | 3 |
| Manslaughter | 4 |
| Uræmic poisoning | 1 |
| Uterus, hemorrhage of | 2 |
| Whooping cough | 4 |
| Total | 566 |

Premature births, 10; Still births, 70. Total 80

COMPARISON.

| | | |
|---|---|---|
| Deaths in month of October, 1874 | | 566 |
| " " September, 1874 | | 809 |
| Decrease | 243 | |
| Deaths in month of October, 1873 | | 644 |
| Decrease | 78 | |

AGES.

| | |
|---|---|
| Under one year | 175 |
| One year to two | 92 |
| Two years to three | 25 |
| Three " " four | 9 |
| Four " " five | 6 |
| Five " " ten | 26 |
| Ten " " twenty | 21 |
| Twenty " " thirty | 18 |
| Thirty years to forty | 44 |
| Forty " " fifty | 43 |
| Fifty " " sixty | 26 |
| Sixty " " seventy | 22 |
| Seventy " " eighty | 19 |
| Eighty " " ninety | 7 |
| Ninety " " one hundred | 3 |
| Total | 566 |

| | |
|---|---|
| Colored | 9 |
| White | 557 |
| Total | 566 |
| Males | 311 |
| Females | 255 |
| Total | 566 |

Married, 157; Single, 409. Total 566

NATIVITIES.

| | |
|---|---|
| Belgium | 2 |
| Bohemia | 6 |
| Canada | 7 |
| Native—Chicago | 89 |
| Foreign, " | 239 |
| United States, other parts | 82 |
| Denmark | 1 |
| England | 8 |
| Germany | 74 |
| Ireland | 59 |
| Italy | 3 |
| Mexico | 1 |
| Norway | 3 |
| Scotland | 5 |
| Sweden | 13 |
| Wales | 1 |
| Unknown | 3 |
| Total | 566 |

Deaths daily, 18¼. Mean temperature, 53.3°. Rain fall, 2.55 inches.

MORTALITY BY WARDS.

| Wards. | No. Deaths. | Pop. in 1874. | Percentage. |
|---|---|---|---|
| 1 | 6 | 5,725 | one death in 954 |
| 2 | 1 | 4,830 | " " 4,830 |
| 3 | 13 | 14,861 | " " 1,143 |
| 4 | 10 | 15,361 | " " 1,536 |
| 5 | 26 | 20,078 | " " 772 |
| 6 | 47 | 35,916 | " " 764 |
| 7 | 59 | 31,722 | " " 538 |
| 8 | 48 | 29,143 | " " 607 |
| 9 | 29 | 31,654 | " " 1,092 |
| 10 | 12 | 17,385 | " " 1,449 |
| 11 | 11 | 14,022 | " " 1,275 |
| 12 | 24 | 16,792 | " " 700 |
| 13 | 13 | 17,892 | " " 1,377 |
| 14 | 24 | 16,720 | " " 697 |
| 15 | 65 | 45,545 | " " 701 |
| 16 | 40 | 21,922 | " " 548 |
| 17 | 36 | 20,777 | " " 327 |
| 18 | 20 | 21,392 | " " 1,069 |
| 19 | 4 | 4,677 | " " 1,169 |
| 20 | 10 | 8,995 | " " 899 |
| | 498 | Ratio of deaths to population in 1874, one death in 699. | |

| | | | |
|---|---|---|---|
| No. deaths in Wards | 498 | Mercy Hospital | 10 |
| Accidents | 14 | Morgue | 1 |
| Bridewell | 1 | Police Station | 1 |
| County Hospital | 11 | St. Luke's Hospital | 2 |
| Foundlings' Home | 19 | Suicides | 3 |
| Hospital for Women and Child'n | 1 | | |
| Industrial Home | 1 | Total | 566 |
| Manslaughter | 4 | | |

